# Pure Testicular Choriocarcinoma with Dermatological, Brain, and Gastrointestinal Metastases

**DOI:** 10.7759/cureus.3083

**Published:** 2018-08-01

**Authors:** Rama Nassri, Mayssan Muftah, Ammar Nassri, Ahmad Alkhasawneh, Jadranko Corak

**Affiliations:** 1 Department of Medicine, Alfaisal University School of Medicine, Dhahran, SAU; 2 Department of Internal Medicine, Emory University School of Medicine, Atlanta , USA; 3 Department of Medicine, University of Florida-Jacksonville, Jacksonville, USA; 4 Department of Pathology, University of Florida College of Medicine-Jacksonville, Jacksonville, USA; 5 Department of Internal Medicine, Dell Seton Medical Center at The University of Texas, Austin, USA

**Keywords:** pure choriocarcinoma, testicular cancer, dermatological metastases, gastrointestinal metastases, germ cell tumor, brain metastases

## Abstract

Testicular choriocarcinoma is a non-seminomatous germ cell tumor (NSGCT) and is the rarest of all testicular cancers. Nearly all choriocarcinomas can be classified as either pure choriocarcinoma or as a component of a mixed germ cell tumor. Pure testicular choriocarcinoma is extremely aggressive and metastasizes early and extensively. We present a case of testicular cancer that metastasized to the skin, gastrointestinal tract, and brain, and discuss the case in light of the available literature.

## Introduction

Pure testicular choriocarcinoma is a non-seminomatous germ cell tumor (NSGCT) and is the rarest and most aggressive form of testicular cancer. Nearly all choriocarcinomas can be classified as either pure choriocarcinoma or as a component of a mixed germ cell tumor. The peak incidence of testicular choriocarcinoma is at 25-30 years of age [1]. Testicular choriocarcinomas are usually unilateral, have markedly elevated serum beta human chorionic gonadotropin (β-hCG), and because of their proclivity for hematogenous spread, they present with metastatic disease, most commonly to the lungs and liver [2-3]. Many patients may present with rare and unusual sites of metastases, which often portend a poorer prognosis. We present a case with widespread metastases including the skin, gastrointestinal tract, and brain.

## Case presentation

A 37-year-old male with no past medical history presented to the emergency room complaining of a two-week history of an enlarging right testicular mass and a three-day history of aching groin pain. A review of systems at admission was negative. A physical examination revealed diffuse enlargement of the right testicle, which was firm and tender to palpation.

**Figure 1:**
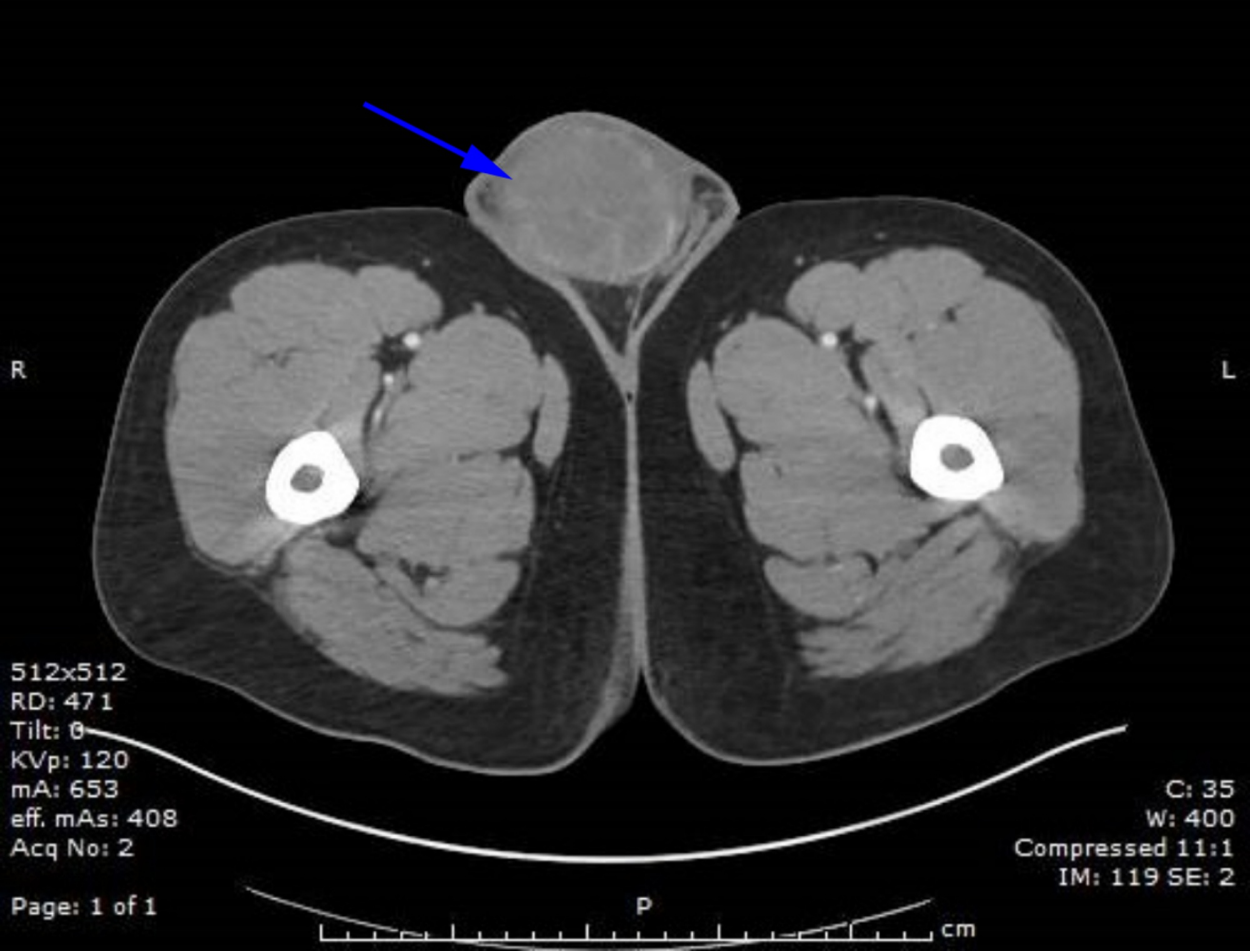
Computed tomography (CT) of the abdomen/pelvis with testicular mass CT of the abdomen and pelvis showing a large right sided testicular mass.

**Figure 2:**
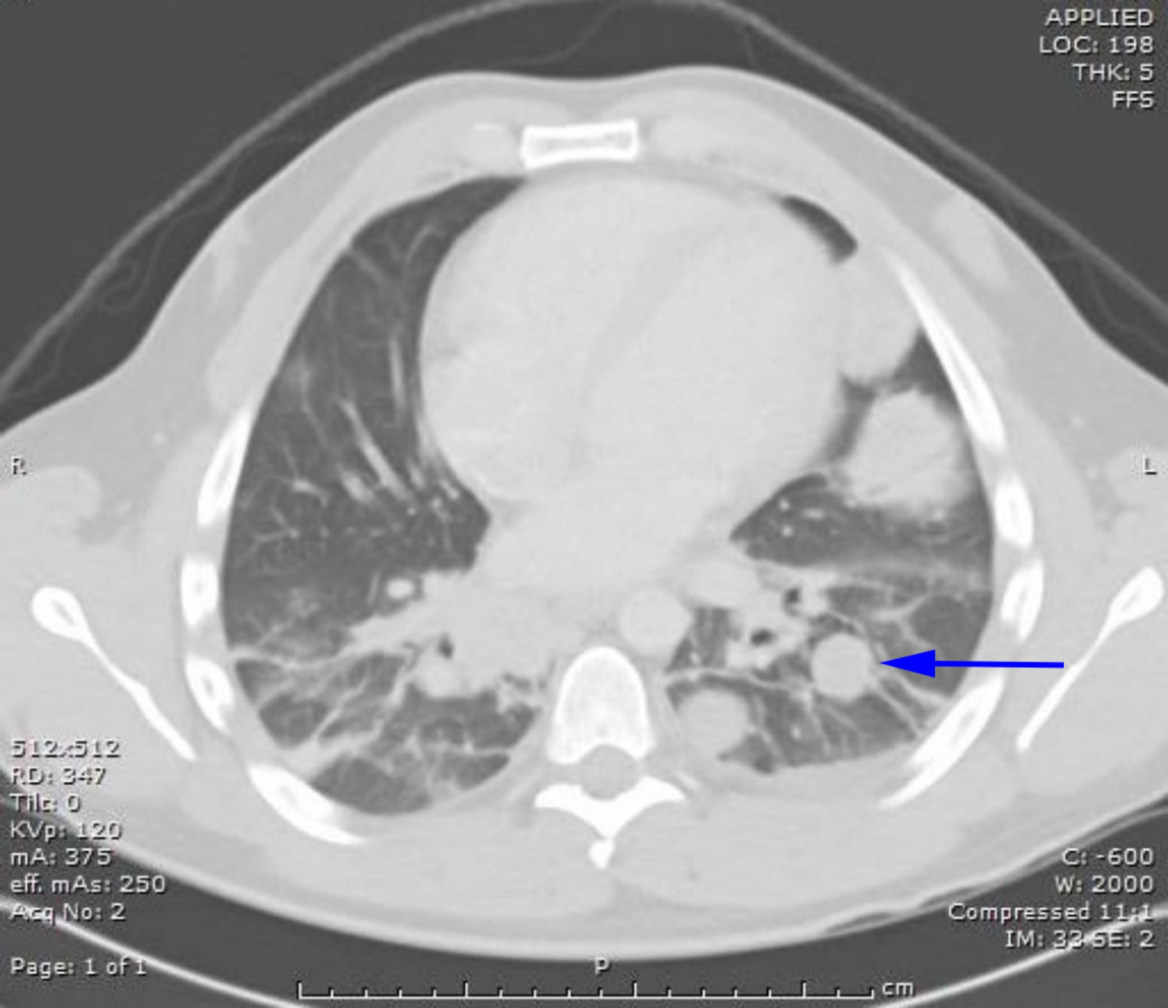
Computed tomography (CT) of the chest with multiple lung metastases CT of the chest showing multiple metastatic lesions in lungs.

Basic laboratory evaluation with a complete blood count (CBC) and complete metabolic panel (CMP) was unremarkable. Lactate dehydrogenase was elevated to 697 U/L, alpha fetoprotein was <2.5 ng/ml and β-hCG was over 278,800 mIU/ml. An ultrasound was performed, which showed an enlarged hypoechoic right testicle measuring 8.9 cm x 5.8 cm x 6.3 cm. A computed tomography (CT) scan of the abdomen and pelvis revealed a right scrotal mass with features consistent with malignancy, nonspecific splenic lesions, retroperitoneal soft tissue implants, and large bilateral pulmonary masses, which were further characterized with a CT scan of the chest (Figures 1-2).

**Figure 3:**
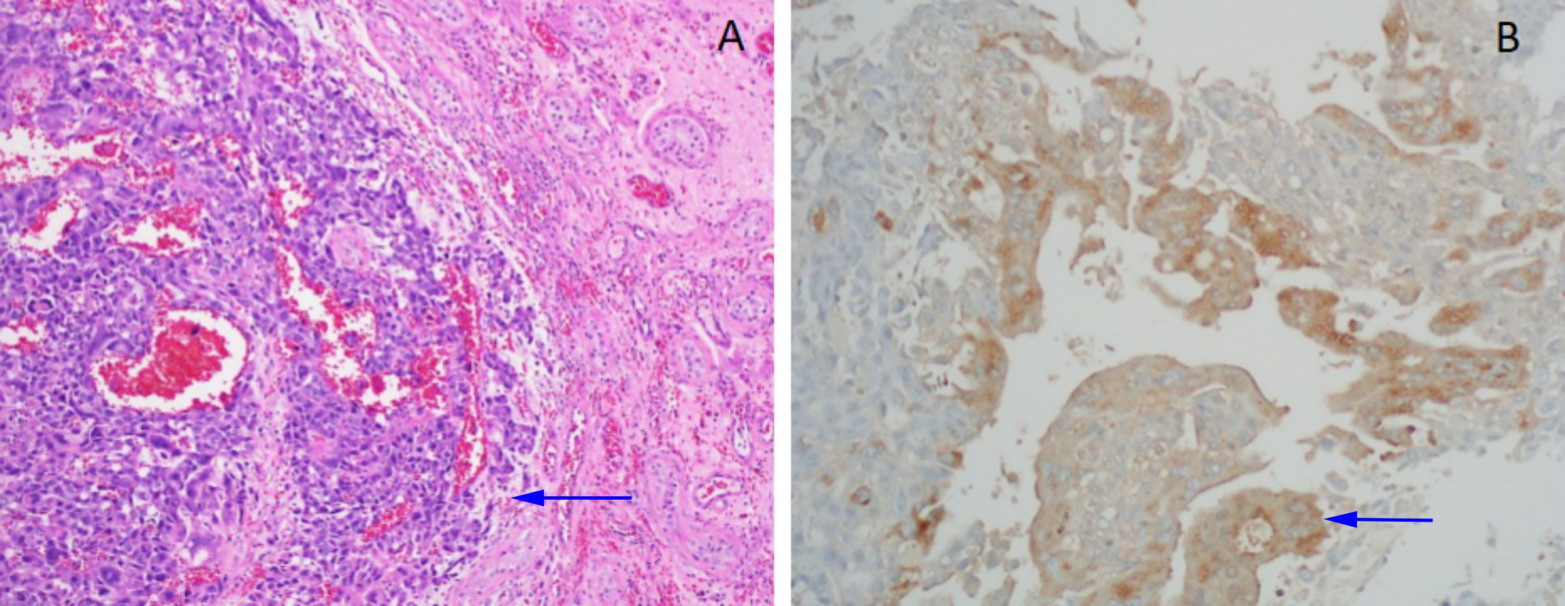
Testicular choriocarcinoma A) Histologic section of the testicular tumor shows sheets of cohesive tumor cells (arrow), and hemorrhage (Hematoxylin and eosin stain 100x). B) Immunohistochemistry for human chorionic gonadotropin (hCG) highlights tumors cells, which supports the diagnosis of choriocarcinoma (arrow, immunohistochemical stain 200x).

Following subspecialty evaluation, the patient underwent a right radical orchiectomy as well as a port-a-cath placement for chemotherapy. Pathology of the testicle revealed 100% choriocarcinoma invading the epididymis as evidenced by extensive parenchymal replacement by biphasic pattern of syncytiotrophoblasts and cytotrophoblasts. β-hCG and keratin AE1/AE3 were positive, and markers CD30, CD117, and OCT4 were negative (Figure 3). It was decided that the patient would be started on bleomycin/etoposide/cisplatin (BEP) therapy, and he was discharged with an appointment to begin treatment as an outpatient.

**Figure 4:**
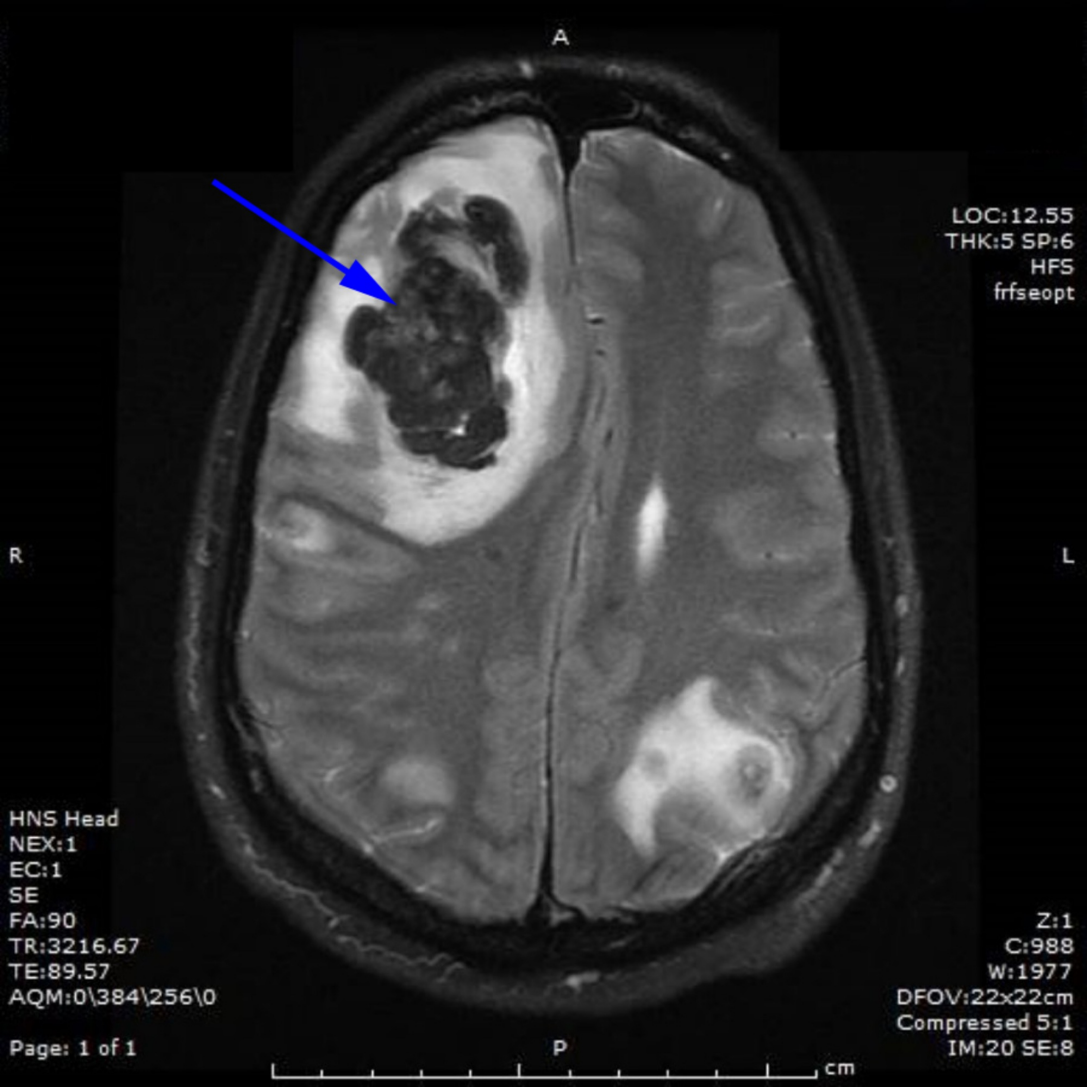
Brain magnetic resonance imaging (MRI) MRI of the brain with 5.5 x 4 cm hemorrhagic mass and midline shift.

Ten days later, the patient was found unconscious at home. According to friends, he had been acting unusual for the past few days, was nauseous, and had several episodes of emesis and one episode of hematemesis. He had also started complaining of a bilateral occipital headache radiating to the frontal region. On physical examination, the patient had a flat affect, was slow to respond, and was acting unusual. A CT scan of the head showed multiple bilateral supratentorial hemorrhagic masses, with the largest mass in the right frontal lobe resulting in midline shift. The patient was admitted to the intensive care unit for close monitoring and treated with mannitol, blood pressure control, and hypertonic saline. Neurosurgery evaluated the patient and decided to hold off on surgical intervention given his intact neurological function. Chemotherapy was then initiated. After one dose of cisplatin, the patient became increasingly altered. Magnetic resonance imaging (MRI) of the brain revealed subfalcine herniation and effacement of the basilar cisterns with slight uncal herniation, in addition to multiple hemorrhagic lesions, the largest of which was in the right frontal lobe (Figure 4). The patient underwent a right frontal craniotomy with removal of the largest mass.

The patient then developed anemia and started having melanotic stool. An esophagogastroduodenoscopy was performed, which showed only mild gastritis and a hiatal hernia. Pathology revealed gastritis and reactive gastropathy. A colonoscopy was noted to have blood in the terminal ileum but was otherwise unremarkable. A push enteroscopy revealed a small ulcerated mass in the jejunum. Two hemoclips were placed there. The enteroscope could not be advanced any further, and it was decided that an angiogram would be performed if the hemoglobin continued to drop or his gastrointestinal bleeding persisted. However, after enteroscopy, his bleeding resolved and hemoglobin levels stabilized.

**Figure 5:**
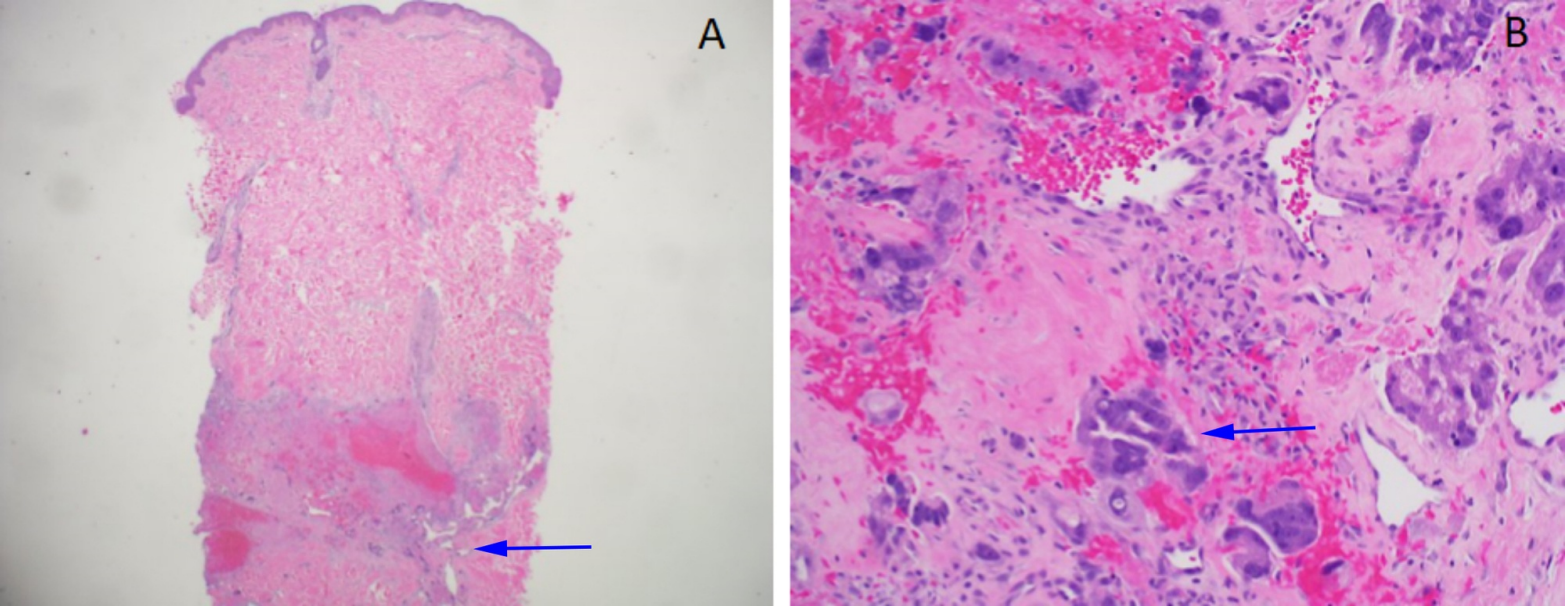
Skin involvement by choriocarcinoma A) Histologic section shows deep dermal involvement by tumor, consistent with metastatic choriocarcinoma (arrow) with hemorrhage (Hematoxylin and eosin stain 40x). B) High power magnification of tumor cells (arrow) with similar morphology to testicular choriocarcinoma (Hematoxylin and eosin stain 200x).

The patient subsequently developed several clustered erythematous-violaceous firm subcutaneous nodules without ulceration or other epidermal changes on the zygoma, with a larger nodule on the anterior neck and right posterior shoulder. A punch biopsy was performed on the shoulder lesion and showed findings consistent with choriocarcinoma with small nests and cords of epithelioid cells with abundant cytoplasm and enlarged highly atypical and pleomorphic nuclei (Figure 5). The patient completed cycle 1 of BEP and then elected to move to be closer to his family.

## Discussion

Testicular cancers are generally divided into germ cell tumors and non-germ cell tumors. Germ cell tumors comprise several cell types and are broadly split into seminoma and non-seminomatous germ cell tumors (NSGCT) with choriocarcinomas being the rarest, comprising 1%-3% of all testicular neoplasms [1]. Nearly all choriocarcinomas can be classified as either pure choriocarcinoma, the rarest of all testicular cancers at 0.1% to 0.8% [3-6]; or as a component of a mixed germ cell tumor, found in approximately 7%-8% of cases [7]. Pure choriocarcinomas are the most aggressive germ cell tumors and metastasize early [1], with half of the patients presenting with metastatic disease, most commonly to the lungs [8].

The characteristic histopathological features of metastatic choriocarcinoma are the presence of cytotrophoblastic and syncytiotrophoblastic cells in the absence of mesenchymal cells. Choriocarcinoma produces high levels of β-hCG, with levels > 50,000 mIU/ml conferring a particularly poor prognosis [9]. Choriocarcinoma as a part of mixed germ cell tumor does not confer a similarly poor prognosis like pure choriocarcinoma because of a decreased propensity for widespread metastases [9].

Cases of pure testicular choriocarcinoma with skin metastases are rare, with only 16 prior reports published [2,10-11]. In general, the skin is a rare site of metastases regardless of cancer type, with estimates of metastases to skin ranging between 0.7%-9% [12]. Approximately 14% of patients with skin metastases have multiple metastatic sites, and the prognosis is considered very poor [12].

Gastrointestinal metastases of choriocarcinoma is also exceedingly rare. Approximately 5% of patients with germ-cell tumors are found to have gastrointestinal metastases [13]; however, metastases from pure choriocarcinoma is much rarer [14-17]. Metastases to the gastrointestinal tract is thought to be either from direct spread from adjacent retroperitoneal lymph nodes or as a result of hematogenous spread. Direct infiltration is more common than hematogenous spread and most commonly involves the small bowel [13,18]. Pure testicular choriocarcinoma with brain metastases has been described 17 times in the literature [19] and provides a dismal prognosis due to the added complications of devastating intracerebral bleeding and herniation.

The treatment approach is similar to other NSGCT with platinum-based therapy recommended as first-line therapy. Case series and reports of pure testicular choriocarcinomas generally report poor prognosis, with a five-year survival rate of less than 80% [1] and some reporting much poorer long-term survival [9,20].

## Conclusions

Pure testicular choriocarcinoma is a rare cancer that is aggressive and often presents with metastatic disease. Skin, gastrointestinal, and brain metastases are all indicators of very poor prognosis. Clinicians should be aware of the myriad presentations of the disease, and prompt and aggressive treatment should be offered to these patients.
